# Robust Framework to Combine Diverse Classifiers Assigning Distributed Confidence to Individual Classifiers at Class Level

**DOI:** 10.1155/2014/492387

**Published:** 2014-09-08

**Authors:** Shehzad Khalid, Sannia Arshad, Sohail Jabbar, Seungmin Rho

**Affiliations:** ^1^Department of Computer Engineering, Bahria University, Islamabad 44000, Pakistan; ^2^Department of Computer Science, COMSATS Institute of Information Technology, Sahiwal, Pakistan; ^3^Department of Computer Science, Bahria University, Islamabad 44000, Pakistan; ^4^Department of Multimedia, Sungkyul University, Anyang-si, Republic of Korea

## Abstract

We have presented a classification framework that combines multiple heterogeneous classifiers in the presence of class label noise. An extension of *m*-Mediods based modeling is presented that generates model of various classes whilst identifying and filtering noisy training data. This noise free data is further used to learn model for other classifiers such as GMM and SVM. A weight learning method is then introduced to learn weights on each class for different classifiers to construct an ensemble. For this purpose, we applied genetic algorithm to search for an optimal weight vector on which classifier ensemble is expected to give the best accuracy. The proposed approach is evaluated on variety of real life datasets. It is also compared with existing standard ensemble techniques such as Adaboost, Bagging, and Random Subspace Methods. Experimental results show the superiority of proposed ensemble method as compared to its competitors, especially in the presence of class label noise and imbalance classes.

## 1. Introduction

In recent years, there has been a growth of research interest in the development of sophisticated data classification techniques as it has many practical applications in variety of fields such as object recognition, surveillance, medical imaging, and financial data analysis. These techniques have been widely used in industry in the form of tools and commercial applications giving so many benefits to them.

Classification of unseen data requires building model of normality of commonly followed patterns that exist in a given domain. Once these models for all the known patterns are learnt, they are then used to classify unseen samples to one of the modelled patterns. A variety of machine learning approaches have been proposed that model the normal pattern and classify newly coming data using the generated models of normality. Statistical approaches for classification [1–3] model a pattern by approximating the density of the training samples belonging to the given pattern. Khalid and Naftel [3] model the pattern by approximating a single multivariate Gaussian for each class. Classification of unseen samples is then performed using Mahalanobis classifier. Classification approaches based on Gaussian Mixture Model (GMM) have also been proposed [4–7]. Various techniques [8–11] based on support vector machines (SVM) have also been presented. The underlying logic behind SVM-based approaches is to identify an optimal hyperplane separating training data belonging to various patterns and then classifying new data based on these identified decision boundaries. One class classifier based on SVM (OCC-SVM) has also been employed for classification and anomaly detection [12, 13]. Neural network based classifiers have also been reported in literature [14, 15]. Owens and Hunter [14] employ self-organizing maps to learn model of normality of given set of patterns. Various *m*-Mediods based approaches have been proposed for data classification and anomaly detection [16–19].

There are many scenarios of distribution of samples belonging to different classes with respect to each other. There may be a high level of overlap in the distributions of samples belonging to different classes. In other cases, samples belonging to various classes may be exhibiting complex shape nonoverlapping distributions with tight decision boundaries amongst them. Similarly, we may have classes that exhibit multivariate distributions of samples within individual patterns. Another commonly occurring phenomenon is that different classes in a dataset have widely varying number of training samples for different classes which is normally referred to as class imbalance problem. This phenomenon is frequent in medical world where the sensitive, dangerous, and later stage of diseases is not very frequent, thus resulting in lesser amount of data available for training our classifiers. On the other hand, nonmalignant forms of these diseases are often commonly occurring and hence the large number of samples is available for training our machine learning algorithms. As general classifiers normally optimize their results by having overall higher classification accuracy, they focus more attention on correctly classifying samples from heavily populated classes as compared to classes which may be sensitive but having lesser number of membership counts. This is in total contrast to our actual expectations. Misclassification of insensitive diseases may be ignored, but doing the same with serious disease classes may result in loss of lives.

The above-mentioned and variety of other scenarios impose challenges for the classifiers. Individual classifiers may be more expert to handle some of these discussed problems, but there is a possibility that it may give poor performance in the presence of others. For instance, GMM is known to perform good in the presence of overlapping distribution of samples belonging to different classes but does not yield good results in the presence of nonoverlapping distributions with tight and complex decision boundaries. On the other hand, SVM performs well in the presence of complex decision surfaces between nonoverlapping distribution, but its performance degrades in the presence of class imbalance problem. Similarly, *m*-Mediods [19] can handle multivariate distribution of samples within a pattern and can handle overlapping distributions belonging to different classes to a reasonable extent.

To overcome this problem, combining classifiers in an ensemble has gained interest quite recently [20–43] in literature. The underlying logic behind classifier ensemble is that many classifiers are combined together in a certain framework to make a final strong classifier whose decision is expected to be more precise and effective as compared to its individual components. The ensemble-based classification approaches can be broadly categorized into two types: homogeneous and heterogeneous classifier ensembles. The homogeneous approaches combine classification algorithms of the same type. Contrary to this, heterogeneous ensemble combines classification algorithms of different types. The most popular and standard ensemble methods include Bagging [30], Boosting (Adaboost) [31], and Random Subspace Methods (RSM) [44] (generalization of Random Forest Method [32]). These are the dominant methods for diversifying and combining classification results.

Most of the existing classification techniques assume that the training data is free of problems such as class label noise and class imbalance problems. There exist some ensemble methods [45–49] that handle class imbalance problem in datasets. There also exist very few methods that cater for the problem of class label noise in the datasets. In this paper, we present a novel classification framework that can handle problems such as class label noise and imbalance classes. An extension of *m*-Mediods based classification approach is proposed that learns the model of normality of classes whilst identifying and filtering training samples representing class label noise. *m*-Mediods classifier is further combined with other well-known classifiers using a proposed framework. We selected GMM and one class classifier based on SVM (OCC-SVM) which are carefully selected based on their capabilities to handle different types of class distributions. The learned models using selected classifiers are then combined by introducing genetic algorithm based weight learning method to learn weights at class level individually for all heterogeneous classifiers. The probabilistic output of each classifier is then combined in a weighted combination at class level to achieve better classification performance. The proposed framework is robust to the presence of class imbalance problem, class label noise, and the presence of multivariate distribution of samples within classes.

The remaining paper is organized as follows. In Section 2, we present a brief review of existing classifier ensemble techniques.  Section 3 presents an overview of the learning of proposed ensemble framework and classification of unseen data using the learned ensemble.  Section 4 presents different classifiers that are used for the construction of proposed ensemble. In Section 5, an extension of *m*-Mediods based approach to filter class label noise is presented. The detailed description of proposed ensemble framework is given in Section 6. Experiments have been conducted to show the effectiveness of proposed approach as compared to the competitors. These experiments are reported in Section 7. The last section summarizes the paper.

## 2. Background and Related Work

Classifier ensembles are known to be very useful methods for improving the classification accuracy as well as diversity. They combine multiple classifiers together to get a single stronger one whose performance is more precise and accurate as compared to its individual members. A variety of factors have been considered in literature to improve the accuracy of the ensemble. These include classifier selection [20, 21, 28, 50], feature selection [21, 27, 32], diversity creation in ensemble of classifiers [34, 39, 40], combination methods [20–23, 25, 30–33, 39], and combining more than one ensemble [24, 41, 42]. Certain ensemble approaches integrate some statistical procedures with them such as Bagging with Principal Component Analysis (PCA) [21], weighting classifier dynamically based on cross validation [22], Dempster-Shafer theory of evidence [25], and supervised projection [26].

Earlier work has put a lot of concentration towards assigning weights to instances as well as classifiers to construct an ensemble by applying weight assignment methods [51] and voting algorithms [29–32, 35, 52, 53]. Bagging [30] and Boosting [31] are one of the most well-known voting and weighting based standard ensemble methods. Breiman [30] introduced the Bagging algorithm which is an independent ensembling method as the output produced by one classifier does not depend on the output of previous classifiers. Bootstrap samples are generated from training set which are obtained by sampling with replacement. A classifier is then learned with different training set in each iteration. It follows the voting approach in order to combine the predictions of classifiers. C.-X. Zhang and J. Zhang [21] combined the concept of Bagging with Principal Component Analysis (PCA) to construct an ensemble. Bauer and Kohavi [52] provided the variant of Bagging, namely, wagging, which makes stochastic assignment of weights to each instance.

Random Forest Method proposed by Breiman [32] uses unpruned decision trees. It consists of a collection of trees like structured classifiers and uses a number of input variables to find the decision at a node of the tree. To classify a new pattern, this algorithm collects votes from every tree in the forest and then uses majority voting to finalize the class label. The generalization of Random Forest approach, referred to as Random Subspace Method (RSM), is also presented [44]. Instead of working only with decision trees, as in the case of Random Forest, RSM can take into account any classifier such as nearest neighbor classifier and support vector machine. A subspace from the feature space representation is identified by randomly selecting a subset of features.

Freund and Schapire proposed Boosting [31] algorithm which enhances the performance of a weak learner by iteratively running it on training data. García-Pedrajas and García-Osorio [26] combine the concept of Boosting with supervised projection method. The technique focuses on misclassified instances as they are used to find supervised projection of data. After getting the projections, the next classifier is trained on it. This approach does not employ majority voting or weighting scheme to combine the classifiers. Merler et al. [53] improve the simple Boosting algorithm by an iterative process which focuses on the inaccuracies produced by the previous classifiers. It entirely concentrates on those samples which are hard to classify. It is a homogeneous and dependent ensemble method. It takes the whole training set in each of its iterations and does not create the bootstrap of samples. Equal weights are initially assigned to every sample in a training dataset. After every iteration, weights of misclassified instances are increased while decreasing the weights of correctly classified ones. It further assigns weight to individual classifier to measure its overall accuracy. The higher weights are given to those classifiers performing accurately. New samples are classified using these weights.

Approaches using dynamic ensemble learning have also been proposed in literature [22, 27]. Zhu et al. [22] provided a new ensemble model named dynamic weighting ensemble classifier based on cross validation (DWEC-CV). In this method, different classifiers are used for different samples. To train and construct a classifier, Random Subspace Method is used. The feature subspaces from the original feature set are selected randomly. The number of feature subspaces selected determines the number of classifiers to be produced. A weight adjusted voting algorithm [29] is introduced which uses a weight vector to weight instances as well as the classifiers. The weight vector, for instance, gives higher weights to those instances which are difficult to classify. On the other side, the classifiers weight vector gives the highest weights to only those classifiers giving better performance on these difficult instances. These classifier weights are identified by those samples having higher weights. DECORATE [54] is a diversity creation method to construct a classifiers ensemble. It creates the additional artificial training data in order to create the diverse classifier ensemble. The methods for dynamic ensemble selection [28] using majority voting rule to combine the classifiers have also been introduced in literature. The methods in [24, 41, 42] combine more than one ensemble in order to achieve improved accuracy and diversity of classifiers. Al-Ani and Deriche [25] combine classifiers using Dempster-Shafer theory.

The problem with majority of exiting ensembles is that they do not cater for the presence of noise in the training data including feature space noise and class label noise, although we expect to have noise related problems in real life datasets. Development of approaches that are robust to various problems such as feature space noise and class label noise has received scant attention. Dietterich [55] performed comparative analysis of standard ensembling techniques in the presence of class label noise. It has been shown that accuracies of these ensembling techniques such as Bagging, Adaboost, and Random Subspace Method degrade in the presence of noise. We will also show through our experimental evaluation that Adaboost, Bagging, and RSM are also not performing well on imbalanced and overlapping classes.

There exist few approaches which cater for the presence of noise in the training data. One of them is a method based on Boosting with supervised projection [26] which shows the noise tolerance of its proposed ensemble method. It compares only one standard ensemble method such as Adaboost with their proposed ensemble using different base learners to prove the sensitivity of Adaboost toward different levels of class label noise. In literature, there exist some researches which show that Boosting methods such as Adaboost degrade their performance when some level of noise is present in the dataset [56]. Arjun and Arora introduced the TRandom [45] Adaboost which performs better than the simple Adaboost in the presence of low and high noise data. In literature there exist classifier methods [46] constructed from the imbalanced datasets. A dataset is said to be imbalanced if the number of instances for all the classes is not equally represented. In literature, ensemble methods [47–49, 57, 58] have been proposed to improve their performance on imbalanced datasets. Zhou et al. [57] provided the relationship between ensemble and its Neural Network components for regression and classification. An EasyEnsemble method and BalanceCascade method have been introduced [47] to handle the class imbalance problem. EasyEnsemble method produces subsets from the majority class and the base learner is trained using each of these subsets and at the end, output of those learners is combined. Ryan and Nitesh [48] gave an extension of Random Subspace Method [44] that joins together SMOTE [46] to overcome the class imbalance problem. In literature, there is a cost sensitive ensemble method for class imbalance dataset [49]. It divides the instances of majority class into several subsets on the basis of imbalanced samples proportions. It then trains the subclassifiers using Adaboost method. Ensemble-based wrapper approach [58] for feature selection from the dataset has also been proposed. This method selects the feature from the data having highly imbalanced class distribution. It creates multiple balanced datasets from the original imbalanced one by doing sampling and then evaluates the feature subsets using ensemble classifiers each trained on balanced dataset.

The contribution of this paper is to present a robust classifier ensemble that combines existing state-of-the-art classifiers such as GMM and OCC-SVM with our proposed *m*-Mediods classifier in a weighted ensemble. A genetic algorithm based weight learning approach to optimize the accuracy of proposed classifier ensemble is presented. The proposed ensemble can handle real life issues of labelled datasets such as class label noise and class imbalance problems.

## 3. Overview of Proposed Ensemble Framework

**Figure 1:**
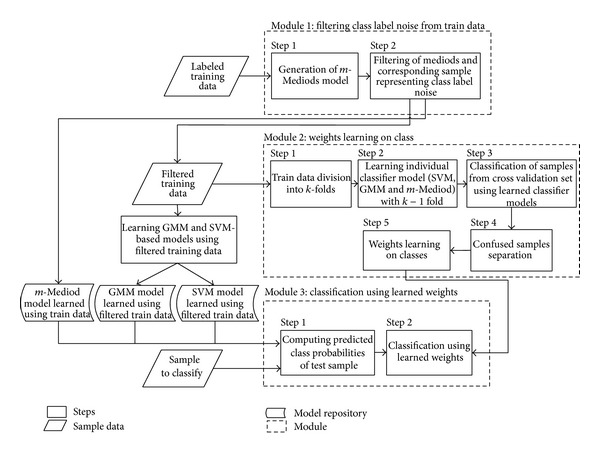
Overview of our proposed framework of classifier ensembles.

Classification in the presence of class label noise and imbalance in the distribution of samples across different classes is a challenging task.  Figure 1 presents an overview of our proposed framework of combining classifiers in an ensemble to handle this challenge. The proposed framework is composed of three main modules: (1) filtering of class label noise from train data to mitigate its effects on model learning process, (2) learning of weights on classes with respect to different classifiers, and (3) classification by combining their probabilistic output using learned weights. The module for filtering class label noise is based on the extension of our previously proposed *m*-Mediods classifier and is composed of two steps. In step 1, we generate the mediods based model to represent different classes. In step 2, we prune those mediods that are isolated or surrounded by mediods from different classes and representing fewer numbers of samples. This module generates the *m*-Mediod based model while filtering the samples representing class label noise. The filtered training data is then further utilized to generate model of normalities of other classifiers. The filtered training data is also used by second module to learn weights on the probabilistic output of classifiers with respect to different classes. We employed *k*-fold cross validation to learn the weights. The models of normality of various classifiers are learned on *k* − 1 fold whereas the left-out fold is used for cross validation. We speed up the learning process by identifying those samples from cross validation set for which there is a confusion; that is, all the classifiers do not predict same class for a given sample. Weight learning using genetic algorithm is then carried out by using only the confused samples. Learning weights at the class level with respect to different classifiers will enable our proposed framework to extract appropriate benefit from the classifier speciality at the class level. A classifier may perform very good while predicting a subset of classes due to existence of a particular type of distribution among those subsets of classes. The same classifier may perform bad for other classes with samples exhibiting different types of distributions. For instance, a classifier performing good in highly overlapping distributions may not work well for nonoverlapping but complex and tight boundaries between them. The learned models of normality of classifiers using filtered training data and weights learned in second module are used by third module for classifying test data. The probabilistic output of each classifier with respect to different classes is combined using learned weights to yield improved classification performance.

## 4. Classifiers

In this section, we describe different classifiers that are used for the construction of proposed classifier ensemble. Given the feature vector representation of training samples from any domain, we propose to generate model of normality using state-of-the-art classifiers including Gaussian Mixture Model (GMM), one class classifier based on support vector machine (OCC-SVM), and an extension of multivariate *m*-Mediods based classifier, as proposed in [19], to incorporate the capacity of handling class label noise. We have selected these classifiers intentionally as they have abilities to handle different types of class distributions. GMM [4] is good in handling overlapping classes and diverse distribution, but it gives poor results with nonoverlapping classes having complex and tight boundaries. On the other hand, SVM [8, 12] handles the problem of complex and tight decision boundaries in a very good manner. It does this by looking for an optimal hyperdimensional decision surfaces which should separate the samples belonging to different classes. However, the effectiveness of OCC-SVM decreases with the increase in the amount of overlap amongst the classes. Another disadvantage of OCC-SVM is that it overlooks those classes having smaller membership counts to correctly classify samples belonging to classes having large membership counts. Multivariate *m*-Mediod [19] classifier has the capacity to cater for the presence of multivariate distribution of samples within a modeled class. It has the strength of modeling complex patterns without imposing any limitation on the shape of distribution of samples within a given pattern. Once the multivariate *m*-Mediods models for all the classes have been learnt, the classification of test samples is achieved using a soft classification technique that can handle for multimodal and overlapping distributions of samples among different patterns within a dataset. It also caters for patterns having small membership count. Combining classifiers with different skills and specialties will enable the ensemble to cover all possible aspects and problems of classification.

One of the major drawbacks of all these classifiers is that their effectiveness degrades significantly with the increasing amount of class label noise in the training data. In the next section, we are presenting an extension of our previously proposed *m*-Mediods based classifier to mitigate the effect of class label noise in training data.

## 5. Modified *m*-Mediods Based Classifier to Filter Class Label Noise

In this section, we are presenting an extension of *m*-Mediods based classifier, as presented in [19], to filter class label noise whilst generating model of normality of different classes. The proposed approach is comprised of three major steps: (1) modelling of known classes using *m*-Mediods model, (2) filtering training samples representing class label noise using *m*-Mediods based model for all the classes, and (3) classification of unseen samples using proposed classifier.

### 5.1. Multivariate *m*-Mediods Based Modeling

Given a labelled training data, we propose to generate *m*-Mediods model that represents the normal class distribution of known classes containing *N* samples using a model composed of *m*-Mediods. Let **S** = {*S*_1_, *S*_2_,…, *S*_*N*_} be our training dataset containing *N* samples. A sample *S*_*i*_ in **S** is represented by a *t*-dimensional feature vector *S*_*i*_ = [*f*_1_, *f*_2_,…, *f*_*t*_]. Let **S** ^(*j*)^ represent the samples belonging to class *j*; an algorithm to generate modified *m*-Mediods based model, whilst filtering samples representing class label noise, is comprised of the following steps.

(1) If the number of samples in training data is less than a threshold *κ*, go to step 8. The value of *κ* is set to a value large enough for which the application of agglomerative merging is feasible with respect to time. We assumed *κ* = 1000.
(2) Initialize the semifuzzy self-organizing map (SFSOM) with a number of output nodes *m*_init_ which is much greater than *m*. Setting higher values of *m*_init_ results in high computational complexity whereas lower values fail to mitigate the problem of local minima associated with quantization. We assumed *m*_init_ = 3∗*m* based on empirical evaluation. Setting much higher values results in increasing computational complexity without having any considerable impact on modeling quality. On the other hand, setting much lower values fails to mitigate the issue of local minima typically affiliated with approaches based on quantization.
(3) Initialize weight vector representation of output nodes, referred to as *W*_*i*_ (where 1 ≤ *i* ≤ *m*_init_), using the probability density function *N*(*μ*, Σ) approximated from training samples in **S** ^(*j*)^.
(4) Determine *k* nearest neighbors **P** of input training sample *S*_*i*_ from set of weight vectors representation of output nodes (**W**) as follows:
  (1) P={P∈W ∣ ∀Q∈P,R∈W−P,    ||Si,Q||≤||Si,R||∧|P|=k},
 where ||·, ·|| is the Euclidean distance function and |·| is the membership count function and *k* = *δ*(*t*), where *δ*(*t*) is the neighborhood size function with respect to current training iteration *t*.
(5) Train the proposed SFSOM network by updating weights in **P** using
  (2) $$\begin{array}{c} W_{c}\left(t+1\right)=W_{c}\left(t\right)+\alpha \left(t\right)\zeta \left(j\right)\left(S_{i}-W_{c}\left(t\right)\right)\;\forall W_{c}\in P, \end{array}$$
 where *W*_*c*_ is the weight vector associated with output neuron *c*, *j* is the order of closeness of *W*_*c*_ to *S*_*i*_  (1 ≤ *j* ≤ *k*), *α*(*t*) is the learning rate of SFSOM with respect to current training cycle *t*, and *ζ*(*j*, *k*) = exp⁡(−(*j* − 1)^2^/2*k* ^2^) is a membership function which has an initial value of 1 and decreases with increasing values of *j*.
(6) Decrease neighborhood size *δ*(*t*) and the learning rate *α*(*t*) with time as follows:
  (3) $$\begin{array}{c} \delta \left(t\right)=\left\lceil \delta_{\text{init}}\left(1-e^{2(t-t_{\max})/t_{\max}}\right)\right\rceil ,\\ \alpha \left(t\right)=1-e^{2(t-t_{\max})/t_{\max}}, \end{array}$$
 where *δ*_init_ is the number of neighbors to be affected when *t* = 1 and *t*_max⁡_ is the maximum number of training iterations.
(7) Iterate through steps from 4 to 6 for all the training iterations.
(8) Compute the membership of training samples by assigning them to the nearest output nodes.
(9) Identify and remove output nodes with zero membership count.
(10) Merge the closest pair of weight vectors, indexed as (*a*, *b*), using the following equation:
  (4) $$\begin{array}{c} W_{ab}=\frac{\left|W_{a}\right|\times W_{a}+\left|W_{b}\right|\times W_{b}}{\left|W_{a}\right|+\left|W_{b}\right|}, \end{array}$$
 where
  (5) (a,b)=argmin⁡(i,j)  ||Wi,Wj||×|Wi|+|Wj|∀i,j∧i≠j.
(11) Iterate through step 10 till the number of output nodes is equal to *m*. Append weight vector **W** to the list of mediods **M** ^(*j*)^ modeling the pattern *j*.
(12) Approximate the density of the local distribution around each mediod by computing the mean of the distance of the mediod from its *k* nearest mediods. Append the average distance to **D** ^(*j*)^ in correspondence with a given mediod in the mediods list **M** ^(*j*)^.

### 5.2. Filtering of Class Label Noise

Once the *m*-Mediods based models of all the classes have been learnt, we apply a filtering process on the complete set of mediods to identify a subset of mediods tentatively representing samples with class label noise. This filtration process is based on the observation that the sample with class label noise will not generally be surrounded by samples belonging to the same class. Consequently, the mediod representing such samples will be surrounded by mediods representing other classes with little or no presence of mediods from the same class. The filtration algorithm to remove samples representing class label noise is composed of the following steps.

(1) Merge sets of mediods **M** ^(*j*)^ modeling different classes *j* into a superset **M** as follows:
  (6) $$\begin{array}{c} M=\left\{M^{\left(1\right)}⋃M^{\left(2\right)}⋃\cdots ⋃M^{\left({\#}_{\text{classes}}\right)}\right\}, \end{array}$$
 where #_classes_ is the total number of classes in a given dataset.
(2) Sequentially select mediod *M*_*i*_ from (**M**). Identify the subset of mediods from **M**, referred to as **P**_*i*_ that are member of *k* nearest neighbors of *M*_*i*_ specified as follows:
  (7) Pi={Pi∈M ∣ ∀Q∈P,R∈M−Pi,  ||Mi,Q||≤||Mi,R||∧|Pi|=k}.
(3) Identify subset of mediods ${\tilde{P}}_{i}$ from **P** that belongs to the same class as *M*_*i*_, specified as follows:
  (8) P~i={P~i∈Pi ∣ ∀Q∈P~i,R∈Pi−P~i,  Γ(Mi)=Γ(Q)∧Γ(Mi)≠Γ(R)},

 where Γ(·) is the function that returns the label of a given sample or mediod.
(4) Prune mediod *M*_*i*_ if there are no mediods in ${\tilde{P}}_{i}$ specified as follows:
  (9) $$\begin{array}{c} M_{\text{noise}}=\left\{M_{i}\in M\;|\;{\tilde{P}}_{i}=\left\{\right\}\right\}\;\forall i. \end{array}$$
(5) Filter mediods representing samples with class label noise using
  (10) $$\begin{array}{c} \hat{M}=\left\{M-M_{\text{noise}}\right\}. \end{array}$$
(6) Filter samples representing class label noise from training data using
  (11) $$\begin{array}{c} \hat{S}=\left\{S_{j}\in S\;|\;S_{j}\in M_{i}\wedge M_{i}\in \hat{M}\right\}\;\forall j \end{array}$$
  (12) P(j)={P(j)∈M(j) ∣ ∀R∈P(j),S∈M(j)−P(j),  ||Q,R||≤||Q,S||∧|P(j)|=k} ∀j.

### 5.3. Multivariate *m*-Mediods Based Classification

Once we have generated the multivariate *m*-Mediods based model of normal classes after mitigating the effect of class label noise, the classification of unseen samples is done by checking the closeness of feature vector representation of unseen sample from the *m*-Mediods models of different classes. The sample is then classified to the class with minimum distance. The proposed algorithm for multivariate *m*-Mediods based classification of unseen samples using learned *m*-Mediods model is composed of the following steps.

(1) Identify *k* nearest neighbors of test sample *Q* from *m*-Mediods model **M** ^(*j*)^ separately for each class *j* as specified in (12).
(2) Compute the membership *I*{*j*} of unseen lesion sample with respect to class *j* as follows:
  (13) $$\begin{array}{c} I\left\{j\right\}=1-\frac{\sum_{j=1}^{k}||Q,P_{j}^{\left(j\right)}||/k}{\bar{D^{\left(j\right)}}}, \end{array}$$
 where $\bar{D^{\left(j\right)}}$ is the average of mean distances corresponding to mediods in **P** ^(*j*)^ as identified in (12). The mean distance corresponding to a given mediod is precomputed and stored in **D** ^(*j*)^ as specified in step 12 of the modeling algorithm, presented in Section 5.1. The test sample is classified to the class with the highest probability (*I*).

## 6. Proposed Classifier Ensemble

In this section, we present a framework for learning and classification using the proposed ensemble of classifiers. Although we intend to use three classifiers as specified in Section 4, we present a generic ensemble framework that can accommodate any number of classifiers. Once the model of different classifiers has been generated, we proposed a genetic algorithm based approach to learn weights on each class for different classifiers using set of identified confused samples. An instance in the population is a weight vector *W* that contains weights to scale the probability/confidence of each classifier on prediction of different classes. The weight vector *W* can be represented as follows:

(14) $$\begin{array}{c} W=\left[\begin{array}{c} w_{C_{1}}^{1},\ldots ,w_{C_{1}}^{j},\ldots ,w_{C_{1}}^{{\#}_{\text{classes}}},\\ \vdots \\ w_{C_{a}}^{1},\ldots ,w_{C_{a}}^{j},\ldots ,w_{C_{a}}^{{\#}_{\text{classes}}},\\ \vdots \\ w_{C_{\Psi }}^{1},\ldots ,w_{C_{\Psi }}^{2},\ldots ,w_{C_{\Psi }}^{{\#}_{\text{classes}}} \end{array}\right], \end{array}$$

where Ψ is the number of classifiers integrated in the proposed framework and *w*_*C*_*a*__^*j*^ is the weight associated with the probabilistic output of classifier *C*_*a*_ regarding class *j*. The algorithm for learning optimal weight values on the classes to improve ensemble accuracy using $\hat{S}$ comprises the following steps.

(1) Initialize the population for genetic algorithm, represented as **W**, by randomly generating #_*W*_ weight vectors. Pad **W** with some predefined weights where a particular classifier gives maximum confidence to one class and no confidence for other classes.
(2) Normalize the weight vectors so that the sum of weights in each vector is equivalent to 1.
(3) Set acc_*W*_ = 0 ∀*W* ∈ **W**.
(4) Divide noise free dataset $\hat{S}$ into *k*-folds. Let *CF*_*l*_ represent the *l*th fold of $\hat{S}$ where *l* = 1,…, *k*.
(5) Select the *l*th fold and treat it as cross validation set ${\hat{S}}_{\text{CV}}^{l}=CF_{l}$. Initialize training set ${\hat{S}}_{\text{train}}^{l}=\{\}$. The training set to learn classifier model is then obtained as follows:
  (15) $$\begin{array}{c} {\hat{S}}_{\text{train}}^{l}=\left\{{\hat{S}}_{\text{train}}^{l}⋃CF_{j}\right\}\;\forall j\wedge j\neq l. \end{array}$$
(6) Learn the individual classifier models such as multivariate *m*-Mediods, GMM, and OCC-SVM using ${\hat{S}}_{\text{train}}^{l}$.
(7) Select a sample *S*_*i*_ from ${\hat{S}}_{\text{CV}}^{l}$ and compute its probability to be classified to different classes using various classifiers as follows:
  (16) $$\begin{array}{c} \text{clas}{\text{s}}_{S_{i}}^{C_{a}}=\arg\underset{\forall j}{\max\;}\text{Pro}{\text{b}}_{C_{a}}\left(y=\text{clas}{\text{s}}_{j}\;|\;S_{i}\right), \end{array}$$
 where Prob_*C*_*a*__(*y* = class_*j*_∣*S*_*i*_) is the probability of a given sample *S*_*i*_ to be classified to class *j* according to classifier *C*_*a*_.

(8) Identify those samples for which at least two classifiers give different class predictions. This is achieved by pruning those samples from ${\hat{S}}_{\text{CV}}^{l}$ for which all the concerned classifiers predict the same class as these will not contribute in weight learning process. Removing such samples will have a positive impact of significantly speeding up the weight learning process on classes. More formally, let pruned cross validation set, containing confused samples, be referred to as $\tilde{S_{\text{CV}}^{l}}$. Set $\tilde{S_{\text{CV}}^{i}}=\{\}$. The filtered cross validation set containing confused samples corresponding to *l*th fold is obtained as specified in
  (17) SCVl~=SCVl~⋃{S∈S^CVl ∣ classSCp≠classSCq  ∀p,q∧p≠q}∀S∈S^CVl.
(9) Repeat steps 5–8 for all the folds.
(10) Select a sample *S* from the set of confused samples corresponding to *i*th fold ($\tilde{S_{\text{CV}}^{i}}$) and compute its probabilities to belong to various classes using different classifiers learnt using ${\hat{S^{i}}}_{\text{train}}$. Combine these probabilities given by different classifiers in an ensemble using a weight vector *W* ∈ **W** as follows:
  (18) $$\begin{array}{c} \text{Pro}{\text{b}}_{\text{ens}}^{j}=\underset{\forall l}{\sum }\omega_{C_{l},j}*\text{Pro}{\text{b}}_{C_{l}}\;\left(y=\text{clas}{\text{s}}_{j}\;|\;S\right)\;\forall j, \end{array}$$
 where *ω*_*C*_*l*_,*j*_ ∈ *W* is the weight assigned to the probabilistic output of sample *S* to belong to class *j* according to classifier *C*_*l*_ and Prob_ens_^*j*^ is the probability of sample *S* to belong to class *j* according to classifier ensemble created using weight vector *W*. Classify the sample using
  (19) $$\begin{array}{c} \text{clas}{\text{s}}_{S}^{C_{\text{ens}}}=\arg\underset{\forall j}{\max}\;\text{Pro}{\text{b}}_{C_{\text{ens}}}\left(y=\text{clas}{\text{s}}_{j}\;|\;S\right). \end{array}$$
(11) Increment acc_*W*_ by 1 if predicted class class_*S*_^*C*_ens_^ is equal to the true label of sample *S*.
(12) Iterate through steps 10-11 for all the confused samples in $\tilde{S_{\text{CV}}^{i}}$.
(13) Iterate steps 10–12 for all the folds in the dataset.
(14) Repeat steps 10–13 ∀*W* ∈ **W** and compute their corresponding accuracies acc_*W*_. The classification accuracies of ensemble computed using different weight vectors in **W** are the objective function that we want to optimize using genetic algorithm.
(15) Generate new sets of weight vectors **W**_new_ by selecting 10 best weight vectors from **W** with respect to their objective function values and applying the genetic operators, that is, crossover and mutation. The objective function using **W**_new_ is computed as specified in steps 10–14. The evolved population of best 20 weight vectors is obtained by selecting the top weight vectors from {**W**⋃**W**_new_}. This step prevents us from losing track of any weight vector that gives us the optimal results during the weight learning procedure whilst filtering the newly evolved but poor population of weight vectors.
(16) Iterate through steps 10–15 till there is no improvement in the optimal classification accuracy of the ensemble for 5 consecutive iterations or the number of iterations over the genetic algorithm exceeds a certain threshold. Select the weight vector *W*_opt_ that yields best accuracy for the learned ensemble.

Once the weight vector to combine classifiers in an ensemble is learned, the classification of test sample *Q* using proposed classifier ensemble is performed as follows:

(20) $$\begin{array}{c} \text{clas}{\text{s}}_{Q}^{C_{\text{ens}}}=\arg\underset{\forall \text{clas}{\text{s}}_{j}}{\max}\;\underset{\forall a}{\sum }\omega_{C_{a},j}*\text{Pro}{\text{b}}_{C_{a}}\left(y=\text{clas}{\text{s}}_{j}\;|\;Q\right)\;\forall j, \end{array}$$

where *ω*_*C*_*a*_,*j*_ ∈ *W*_opt_.

## 7. Experiments

In this section, we present different experiments that are performed to show the effectiveness of proposed approach as compared to the competitors.

### 7.1. Datasets

To evaluate the performance of our proposed classifier ensemble methodology, four real life datasets have been used including IRIS, Satimage, DiaretDB, German, Pima Indian Diabetes, and Heart datasets. A brief overview of these datasets is given in Table 1. The distribution of instances in different classes within a dataset is presented in Table 2 to highlight the variation in distribution of samples among the classes.

### 7.2. Experiment 1: Evaluation of Proposed Classifier Ensemble Approach

The purpose of this experiment is to assess the effectiveness of our proposed ensemble approach as compared to individual classifiers in the ensemble. The experiment is conducted on DiaretDB dataset. We have extracted 70% of the instances separately from each class and treated it as a training data. The remaining 30% of the samples from each class are treated as test data. We employ *k*-fold cross validation with *k* = 7 to learn the classifier models individually in an ensemble as specified in Section 6. We managed to extract only 159 confused samples out of 2156 samples present in training DiaretDB dataset. Selection of only confused samples from the train dataset speeds up the weight learning process on all individual classes present in the dataset. Learning of optimal weights on classes for the proposed ensemble is done as specified in Section 6. The classification accuracies of classifier ensemble approach and its individual classifier members, using test dataset, are presented in Table 3. It is observed that hybrid classifier yields the best accuracy, that is, 99.675%, as compared to its individual member classifiers which shows the effectiveness of our proposed classifier ensemble as compared to individual classifiers.

### 7.3. Experiment 2: Comparison of Proposed Classifier Ensemble Approach with Competitors

This experiment is conducted to compare the performance of proposed ensemble method as compared to the competitors including Adaboost, Bagging, and Random Subspace Method (RSM). We have performed the experiment using DiaretDB, IRIS, Satellite Image, Heart, Pima Indian Diabetes, and German datasets. The experimental setup is similar to the one specified in Experiment 1. The competitive approaches require the manual specification of number of iterations used for training the ensemble. We use different number of iterations, that is, 50, 100, and 150 iterations, to learn the competitive learning approaches. The classification accuracies of competitors for variety of real life datasets, using 50, 100, and 150 iterations, are reported in Table 4. We can observe from the table that the performance of competitors is affected by changing the number of iterations. Adaboost shows more sensitivity toward the selection of the number of iterations followed by Bagging and RSM. We select the best classification accuracies of different competitors with respect to different number of iterations (represented in bold in Table 4) and used them to compare it with the classification accuracies obtained using our proposed ensembling approach. The results are presented in Table 5. As obvious from Table 5, the proposed approach gives the best classification accuracy results as compared to the competitors. Our approach is also independent of the manual specification of number of iterations.

### 7.4. Experiment 3: Comparing Proposed Ensemble Approach with Competitors in the Presence of Noise

The purpose of this experiment is to study the sensitivity of proposed ensemble approach and its competitors in the presence of class label noise. Class label noise is induced by randomly changing the label information of certain number of samples in training data with wrong labels. To simulate different noise level, we simulated noise in 5% and 10% of samples in training data. The remaining setup of the experiment is the same as specified in Experiment 2. The comparison of proposed approach with competitors using DiaretDB, IRIS, Satimage, Pima Indian Diabetes, and German datasets with class label noise is presented in Table 6. Based on these results and their comparison with performance of various approaches without the presence of class label noise, as presented in Table 5, we can see that the performance of Adaboost is affected by the presence of class label noise specially for Satimage and German data. With DiaretDB, the performance of Adaboost goes down for 10% noise level. Bagging is relatively less sensitive to noise than Adaboost. RSM also shows sensitivity to various noise levels using different dataset. However, it can be observed from Table 6 that our proposed ensemble approach shows the least sensitivity toward class label noise as compared to its competitors. Filtering class label noise using proposed extension of *m*-Mediods based modeling approach has significantly mitigated the effect of class label noise. Resultantly, the proposed approach gives more accurate results as compared to competitors in the presence of class label noise.

### 7.5. Experiment 4: Analyzing Sensitivity of Ensembling Approaches to Class Imbalance in Datasets

The performance of proposed ensemble approach in the presence of class imbalance problem is evaluated and compared in this experiment. The experimental setup is similar to the one specified in Experiment 2. The confusion matrices of competitors and proposed approach using DiaretDB, Satimage, Heart, Pima Indian Diabetes, and German datasets are presented in Tables 7, 8, 9, 10, and 11, respectively. Looking at the confusion matrices obtained using different ensembling approaches across variety of datasets, it is obvious that Adaboost is performing poorly on class imbalance problem. Adaboost attempts to increase overall classification accuracy by focusing on classes with large membership count and ignoring those with lesser memberships. This is obvious from results for class 3 and class 4 for DiaretDB dataset, as presented in Table 7(a). Similarly, we can observe the same phenomena with classes 3, 4, and 6 of Satimage dataset as presented in Table 8(a). Classes 3 and 6 have large number of samples and hence they have overshadowed class 4 which has resulted in misclassification of many class 4 samples to class 3 and class 6. Similarly, the same situation is observed with Pima Indian Diabetes and German datasets as all of these binary class datasets have imbalanced number of instances in both classes. It is observed that there is a large difference between both classes of each of these two datasets. From Table 10(a) it can be observed that Adaboost performs well on the class having large membership count such as class 1, but its performance goes down on the class comprising small number of instances. It also shows poor performance on class 1 of German dataset having very small membership count as presented in Table 11(a). On the other hand, Bagging and RSM perform better than Adaboost but are still affected by the presence of class imbalance issue. The reason behind this behavior of competitors is that they focus on improving their overall classification accuracy without considering individual classes irrespective of their membership counts. As compared to competitors, proposed ensemble approach based on weight learning on classes is performing well on DiaretDB, Satimage, Heart, Pima Indian Diabetes, and German datasets as shown in Tables 7(d), 8(d), 9(d), 10(d), and 11(d), respectively.

### 7.6. Discussion

In this section, we presented a variety of experiments to demonstrate the superiority of proposed approach as compared to competitors in the presence of real world problems including presence of different level of class label noise class imbalance problem. The experimental results on different datasets obtained from variety of domains are presented. It has been observed that the proposed approach performs better than the existing state-of-the-art approaches such as Adaboost, RSM, and Bagging. The results presented in Table 4 demonstrated that the proposed approach gives the best classification accuracy results as compared to the competitors without imposing any requirement of manual specification of number of iterations. The proposed framework for combining classifiers is also robust to the presence of different level of class label noises. The proposed approach is least affected by the presence of noise as highlighted in Table 6. Robustness of proposed approach to the presence of class imbalance problem is highlighted in Experiment 4. This superiority of proposed approach, as compared to competitors, is attributed to the selection of classifiers with different specialties to handle various types of distribution that is expected within a dataset. Learning weights on these classifiers at the class level will result in having major contribution of decision making from a classifier that suits the localized probability distribution. This results in significant reduction of false positives and false negatives as compared to the competitors.

## 8. Conclusion

In this paper, we have discussed the issue of combining classifiers in an ensemble to enhance the overall classification accuracies. A novel classifier ensemble framework is presented that combines heterogeneous classifiers while handling the problems of class label noise and class imbalance problem. An extension of *m*-Mediods based approach is presented to handle and filter samples in training data that are expected to have incorrect labels. The filtered data is then used to learn the remaining classifier models. These individual models are further combined by introducing weight learning method to learn weights on classes for each individual classifier to construct an ensemble. A genetic algorithm based approach is also presented that quickly searches for an optimal set of weights at class level for different classifiers. The weighted classifier ensemble is then used to classify unseen samples to one of the known classes.

Experiments are conducted to compare the performance of proposed classifier ensemble framework with existing state-of-the-art approaches such as Adaboost, Bagging, and RSM. It has been shown that the proposed classifier ensemble gives better classification accuracies, as compared to the competitors, on variety of datasets selected from different domains. Experimental results to show the robustness of proposed approach to class label noise are also presented in Section 7.4. The proposed approach gives better classification results than competitors in the presence of various levels of class label noise as obvious from Table 6. The sensitivity of proposed approach, as compared to competitors, in the presence of class imbalance problem is also analyzed. The proposed approach shows the least sensitiveness to class imbalance issue as demonstrated in Tables 7–9, respectively.

## Figures and Tables

**Table 1:** Overview of datasets used for experimental evaluation.

Dataset	Description	Number of samples	Number of features	Number of classes
IRIS	A flower dataset [59] used as a test bed for comparison of classification techniques.	150	4	3

DiaretDB	A lesion image dataset [51] used for diabetic retinopathy detection methods. These images are processed by [60, 61] to extract feature vector representation of lesions.	3080	15	4

Satimage	A publicly available Satellite Images dataset [59] generated from Landsat scanner image data.	6435	36	6

Heart	A good dataset [59] to test the ML algorithms. In this dataset, each patient is classified as normal and abnormal.	267	23	2

Pima Indian diabetes	It is provided by National Institute of Diabetes and Digestive and Kidney diseases [59]. It is a dataset of all female patients, at least 21 years old.	768	8	2

German	This is a German credit dataset [59].	1000	20	2

**Table 2:** Description of number of instances present in each class of datasets.

Dataset	Class 1	Class 2	Class 3	Class 4	Class 5	Class 6
IRIS	50	50	50			
DiaretDB	1028	725	1262	65		
Satimage	1533	703	1358	626	707	1508
Heart	55	212				
Pima Indian Diabetes	500	268				
German	300	700				

**Table 3:** Classification accuracies of proposed classifier ensemble as compared to individual classifiers using DiaretDB dataset.

Method	% accuracy
Proposed classifier ensemble	**99.675**
Modified *m*-Mediods	97.62
GMM	98.59
OCC-SVM	93.72

**Table 4:** Classification accuracies of competitors using different number of training iterations.

Dataset	Adaboost			Bagging			RSM		
	Iter = 50	Iter = 100	Iter = 150	Iter = 50	Iter = 100	Iter = 150	Iter = 50	Iter = 100	Iter = 150
IRIS	**100**	97.8	97.8	**100**	97.8	100	**100**	**100**	**100**
DiaretDB	91.92	**92.35**	**92.35**	97.68	**97.9**	97.68	**97.24**	97.1	97
Satimage	76.1	77.1	**78.1**	91.25	91.5	**91.7**	**90.99**	90	90
Heart	**67.5**	**67.5**	66.3	62.5	62.5	**63.8**	**68.8**	**68.8**	**68.8**
Pima Indian Diabetes	75.37	74.08	**76.53**	77.48	77.51	**78.01**	76.34	**77.6**	76.34
German	72	**72.33**	71.1	**73.1**	72.4	72.67	**72.7**	72.3	72

**Table 5:** Classification accuracies of competitors using different number of training iterations.

Dataset	Adaboost	Bagging	RSM	Proposed approach
IRIS	**100**	**100**	**100**	**100**
DiaretDB	92.35	97.9	97.24	**99.675**
Satimage	78.1	91.7	90.99	**94.05**
Heart	67.5	63.8	68.8	**70.27**
Pima Indian Diabetes	76.53	78.01	77.6	**78.26**
German	72.33	73.1	72.7	**74.7**

**Table 6:** Comparison based on classification accuracies of proposed approach as compared to competitors in the presence of different levels of class label noise.

Dataset	Adaboost		Bagging		RSM		Proposed approach	
	5% noise	10% noise	5% noise	10% noise	5% noise	10% noise	5% noise	10% noise
IRIS	**98**	**97.8**	95.6	95.6	97.6	97.6	**98**	**97.8**
DiaretDB	90.95	88.6	97.2	96.1	95.9	94.1	**97.9**	**97.2**
Satimage	75.5	72.2	91.2	90.8	90.5	90.3	**93.4**	**92.6**
Pima Indian Diabetes	75.4	74.9	77.3	76.1	76.7	74.5	**78**	**77.7**
German	71.58	69.9	72.6	71.3	69.9	68.4	**73.6**	**72.8**

**(a):** 

	*C*1	*C*2	*C*3	*C*4
*C*1	284	14	10	0
*C*2	13	203	2	0
*C*3	2	10	367	0
*C*4	0	2	17	0

**(b):** 

	*C*1	*C*2	*C*3	*C*4
*C*1	307	0	1	0
*C*2	2	215	1	0
*C*3	2	3	374	0
*C*4	0	2	8	9

**(c):** 

	*C*1	*C*2	*C*3	*C*4
*C*1	305	0	3	0
*C*2	5	212	1	0
*C*3	1	0	378	0
*C*4	0	2	13	4

**(d):** 

	*C*1	*C*2	*C*3	*C*4
*C*1	306	1	1	0
*C*2	2	217	1	0
*C*3	0	0	379	0
*C*4	0	0	0	19

**(a):** 

	*C*1	*C*2	*C*3	*C*4	*C*5	*C*6
*C*1	363	0	22	2	4	69
*C*2	13	177	0	2	13	6
*C*3	2	0	394	5	0	6
*C*4	4	0	60	14	0	110
*C*5	30	2	1	3	137	39
*C*6	1	0	17	11	2	421

**(b):** 

	*C*1	*C*2	*C*3	*C*4	*C*5	*C*6
*C*1	446	1	9	0	4	0
*C*2	0	206	0	3	1	1
*C*3	2	2	391	10	0	2
*C*4	2	1	34	120	1	30
*C*5	9	2	0	0	188	13
*C*6	0	2	6	21	5	418

**(c):** 

	*C*1	*C*2	*C*3	*C*4	*C*5	*C*6
*C*1	450	1	9	0	0	0
*C*2	0	255	0	2	3	1
*C*3	1	3	390	8	0	5
*C*4	1	1	34	120	1	30
*C*5	13	2	0	1	177	19
*C*6	0	1	7	27	4	413

**(d):** 

	*C*1	*C*2	*C*3	*C*4	*C*5	*C*6
*C*1	448	2	10	0	0	0
*C*2	1	204	0	1	5	0
*C*3	1	0	393	8	4	1
*C*4	1	0	12	153	14	8
*C*5	1	0	1	6	202	2
*C*6	0	1	3	11	23	414

**(a):** 

	*C*1	*C*2
*C*1	16	17
*C*2	9	38

**(b):** 

	*C*1	*C*2
*C*1	12	21
*C*2	8	39

**(c):** 

	*C*1	*C*2
*C*1	13	20
*C*2	5	42

**(d):** 

	*C*1	*C*2
*C*1	10	23
*C*2	1	46

**(a):** 

	*C*1	*C*2
*C*1	128	22
*C*2	51	29

**(b):** 

	*C*1	*C*2
*C*1	135	15
*C*2	39	41

**(c):** 

	*C*1	*C*2
*C*1	131	19
*C*2	38	42

**(d):** 

	*C*1	*C*2
*C*1	138	12
*C*2	27	53

**(a):** 

	*C*1	*C*2
*C*1	34	56
*C*2	27	183

**(b):** 

	*C*1	*C*2
*C*1	28	62
*C*2	5	205

**(c):** 

	*C*1	*C*2
*C*1	28	62
*C*2	8	202

**(d):** 

	*C*1	*C*2
*C*1	39	51
*C*2	3	207
